# Effects of Lavender Flower Distillation Residue Powder on Performance, Egg Quality and Yolk Antioxidant Capacity in Laying Japanese Quail

**DOI:** 10.3390/ani16060876

**Published:** 2026-03-11

**Authors:** Osman Olgun, Gözde Kılınç, Ainhoa Sarmiento-García

**Affiliations:** 1 Department of Animal Science, Agriculture Faculty, Selcuk University, 42130 Konya, Türkiye; oolgun@selcuk.edu.tr; 2 Department of Food Processing, Suluova Vocational Schools, Amasya University, 05500 Amasya, Türkiye; gozde.kilinc@amasya.edu.tr; 3 Departamento de Producción Animal, Facultad de Veterinaria, Universidad de León, 24007 León, Spain

**Keywords:** egg quality, lavender by-product, oxidative status, performance, quail

## Abstract

This study evaluated the effects of lavender flower distillation residue powder (LRP) as a dietary supplement on performance, egg quality and yolk oxidative status in laying Japanese quail. Seventy-five 24-week-old hens were allocated to three dietary treatments (0, 1 or 2 g/kg residue powder) for 10 weeks, with five replicate cages per treatment and five birds per cage. Feed intake increased at the inclusion level of 1 g/kg but decreased at 2 g/kg compared with the control group (*p* < 0.01), whereas egg production, egg weight, egg mass and feed conversion ratio were not affected (*p* > 0.05). Dietary supplementation reduced the proportion of damaged eggs (*p* < 0.05) and increased eggshell breaking strength (*p* < 0.01) in a dose-dependent manner. Yolk redness (a*) and Roche color score were higher in birds fed 1 g/kg of LRP (*p* < 0.01). Yolk radical-scavenging capacity (DPPH) increased at 1 g/kg (*p* < 0.01), while yolk malondialdehyde (MDA) concentration decreased with increasing inclusion level (*p* < 0.01). Overall, LRP may be used as a natural feed additive to improve eggshell integrity, yolk color and yolk antioxidant status in laying quail; however, the inclusion level should be carefully considered due to its contrasting effects on feed intake.

## Simple Summary

The use of agricultural by-products in animal nutrition is gaining interest as a way to reduce waste, lower feeding costs and improve the quality of animal products. Lavender is a well-known aromatic plant that contains natural compounds with beneficial properties, and its residues obtained after essential oil extraction are inexpensive and widely available. This study explored whether lavender flower distillation residue powder (LRP) could be used in the diet of laying quail. The results suggest that including small amounts of this by-product in quail diets can improve eggshell quality, enhance yolk color and increase the antioxidant protection of egg yolk, without negatively affecting egg production. A moderate inclusion level (1 g/kg of feed) was associated with increased feed intake, whereas a higher-level reduced appetite. Overall, these findings indicate that LRP could be a valuable and sustainable feed ingredient, contributing to better egg quality while supporting the reuse of agricultural by-products.

## 1. Introduction

The use of plant-derived products in animal nutrition has increased in recent years, driven by the search for natural feed additives capable of improving animal performance, product quality and oxidative stability, while supporting more sustainable production systems [1,2,3]. Aromatic and medicinal plants are of particular interest due to their content of bioactive compounds with antioxidant, antimicrobial and anti-inflammatory properties, which may contribute to animal health and the quality of animal-derived foods [4].

Lavender (*Lavandula angustifolia*), a flowering plant in the Lamiaceae family, is widely recognized for its aromatic and bioactive properties and has been traditionally used in a variety of industrial and agricultural applications [5]. Owing to its rich phytochemical composition, lavender has attracted increasing scientific interest in animal nutrition, particularly as a natural source of bioactive compounds with antimicrobial [6,7] and antioxidant activities [8,9], which are relevant for improving animal health and the quality of animal-derived products.

The biological activity of lavender is largely attributed to its volatile and phenolic constituents. Consequently, most nutritional studies have focused on the use of lavender essential oil as a dietary supplement in poultry. In laying birds, supplementation with lavender essential oil has been reported to improve performance and egg quality parameters in some studies [10,11,12], whereas other studies reported no significant differences compared with control diets [13]. In broilers, dietary inclusion of lavender essential oil has been more consistently associated with improvements in body weight and feed conversion ratio [5,14,15,16]. Moreover, several studies have demonstrated that lavender essential oil supplementation can enhance antioxidant status in serum [15,17], eggs [11] and meat [18]. The variability observed among studies may be partly related to differences in the form of lavender used, the volatility of essential oil constituents and their bioavailability. In contrast to essential oil, which mainly contains volatile compounds, lavender flower distillation residues retain non-volatile phenolic constituents after the extraction process and represent an underutilized agro-industrial by-product [19,20,21,22]. Evaluating these residues may therefore help to clarify the functional potential of lavender-derived products. However, information regarding the use of lavender distillation residues in poultry nutrition, particularly in laying birds and with respect to egg quality and yolk oxidative stability, remains scarce.

The valorization of agro-industrial by-products has gained increasing attention as part of sustainability and circular economy strategies, particularly for their potential use as functional feed ingredients [2,3]. A wide range of agricultural by-products, such as purple carrot [1], lemon peel [2], red cabbage [3], among others, have been evaluated in laying poultry diets due to their functional properties and potential benefits for egg quality and oxidative stability. Lavender is primarily cultivated for the extraction of its essential oil, which is widely used in pharmacology and cosmetics [19] and represents only a small fraction of the plant biomass, accounting for approximately 2–10% of the harvested material [20,21,22]. Consequently, the distillation process generates substantial amounts of residual plant material. Lavender flower distillation residues are rich in non-volatile phenolic acids (e.g., rosmarinic, caffeic, p-coumaric and neochlorogenic acids) and flavonoids (e.g., quercetin derivatives and myricetin), which are known to contribute to antioxidant activity and may influence yolk oxidative stability and pigmentation [23,24]. In addition to these phytochemicals, lavender residue contains minerals, fats, proteins and other nutrients that may be utilized by farm animals [23,25,26].

Japanese quail (*Coturnix japonica*) are widely used both as a commercial egg-producing species and as an experimental model in nutritional studies due to their high laying rate, rapid turnover and sensitivity to dietary interventions [1,2,3]. These characteristics make them particularly suitable for evaluating the effects of dietary bioactive compounds on egg quality traits and yolk oxidative status, which are the main objectives of the present study.

To our knowledge, there are no published studies evaluating the use of lavender flower distillation residue powder (LRP) as a feed additive in poultry nutrition. In contrast to the extensive literature on lavender essential oil [5,10,11,12,13,14,15,16,17,18], this study focuses on LRP, an underexplored by-product retaining non-volatile bioactive compounds. The present findings provide novel evidence supporting its potential use as a functional feed additive in laying quails, while simultaneously contributing to the valorization of agro-industrial residues in line with circular economy principles and sustainable poultry production. Given the nutritional and phytochemical composition of LRP, this by-product represents a promising candidate for use as a functional feed ingredient in poultry diets. Therefore, the present study aimed to evaluate the effects of dietary supplementation with LRP on performance parameters, egg quality characteristics and yolk antioxidant status in laying Japanese quail.

## 2. Materials and Methods

### 2.1. Ethical Statement

The study involved a non-invasive dietary intervention conducted under standard farming conditions. According to institutional regulations, the experimental protocol was reviewed and approved by the Dean of the Faculty, who confirmed that no formal authorization from an ethics committee was required for this type of study. All procedures complied with European legislation on the protection of animals used for scientific purposes (Directive 2010/63/EU). No experimental procedures expected to cause pain, suffering or distress beyond routine husbandry practices were applied. Animals were monitored daily for general health status, behavior and feed and water intake. The study protocol was defined before the experiment, but was not preregistered in a public database.

### 2.2. Animals, Housing and Experimental Design

The experiment was carried out at an indoor experimental facility located in Selçuklu, Konya, Türkiye (38°01′36″ N, 32°30′45″ E). A total of 75 laying Japanese quail (*Coturnix japonica*), 24 weeks of age, were used in the experiment. Inclusion criteria were defined a priori and required that all birds were clinically healthy at the start of the experiment and managed under standard commercial husbandry conditions. A priori exclusion criteria included the removal of any animal showing signs of illness or compromised welfare during the experimental period. However, no animals met these exclusion criteria during the study. Consequently, no animals, experimental units or data points were excluded, and all collected data were included in the statistical analyses.

At the beginning of the experimental period, quail were individually weighed to ensure homogeneity among groups (270.0 ± 9.2 g) and were randomly allocated to one of three dietary treatments. Each treatment consisted of five replicate cages, with five birds per cage, resulting in 25 birds per treatment. Cages were randomly allocated to dietary treatments at the beginning of the study. The randomization sequence was generated using a simple random allocation procedure, whereby cages were assigned to treatments using a random number generator. Blinding was not feasible due to the nature of the dietary treatments. Researchers involved in animal management, outcome assessment and data analysis were aware of group allocation throughout the study.

The experimental period lasted 10 weeks. During this time, birds were fed one of three experimental diets: a control diet without LRP and two diets supplemented with LRP at inclusion levels of 1 or 2 g/kg of feed. The number of birds and replicates per treatment was selected in accordance with previous nutritional studies conducted in laying quail, which have demonstrated that this experimental design provides adequate sensitivity to detect differences in performance and egg quality parameters [1,2]. Throughout the study, birds were maintained under controlled environmental conditions, with a photoperiod of 16 h of light and 8 h of darkness. Housing consisted of sanitized wire-mesh cages (30 cm × 45 cm) arranged in a well-ventilated room maintained at a constant ambient temperature of 20 ± 2 °C. Feed and water were provided ad libitum, and birds were monitored daily for general health status, behavior and feed and water intake. To minimize potential cage location effects, replicate cages from each dietary treatment were distributed across the facility rather than being grouped in a single area.

### 2.3. Diets and Supplementation

Three experimental diets were formulated for the study. A basal diet was formulated to meet the nutritional requirements of laying Japanese quail, as recommended by the National Research Council [27], and was used as the control diet. Two additional diets were prepared by supplementing the basal diet with LRP at inclusion levels of 1 or 2 g/kg of feed.

Lavender flower distillation residue was obtained after essential oil extraction from *Lavandula angustifolia* flowers and was supplied by the Distillation Unit of Suluova Vocational Schools of Amasya University, Türkiye. Lavender flower distillation residue was obtained after the essential oil extraction process. Following distillation, the residual plant material was dried at 40–45 °C, ground to obtain a fine powder and stored under dry conditions until incorporation into the experimental diets. The residue powder was then thoroughly mixed with the basal diet to ensure homogeneous distribution. All experimental diets were prepared before the start of the trial and were offered to birds in mash form throughout the experimental period.

Lavender flower distillation residue powder was used as a feed additive at low inclusion levels to evaluate its functional and antioxidant properties. The experimental diets were formulated to be isoenergetic and isonitrogenous, with the only difference among treatments being the level of LRP. Samples of LRP were analyzed to characterize their phenolic content and antioxidant capacity before dietary inclusion (Table 1). Total phenolic content was determined using the Folin–Ciocalteu method, following the procedure described by Singleton and Rossi [28]. Results were expressed as gallic acid equivalents. The antioxidant capacity of the lavender residue powder was assessed by measuring its reducing power, expressed as a percentage, using a spectrophotometric method as described in the corresponding protocol. All analyses were performed in duplicate under standard laboratory conditions.

The proximate composition of the experimental diets was determined according to official AOAC International methods [29] and expressed on an as-fed basis. Crude protein content was analyzed using the Kjeldahl method (AOAC 990.03), ether extract was quantified by Soxhlet extraction (AOAC 2003.06), moisture content was determined by oven-drying at 105 °C (AOAC 2001.12), and ash content was measured by incineration in a muffle furnace. Detailed ingredient composition and analyzed nutrient content of the diets are presented in Table 2.

### 2.4. Productive Performance Measurements

Productive performance parameters were recorded to evaluate the effects of dietary supplementation with LRP. Body weight of individual birds was measured at the beginning and at the end of the experimental period using a precision scale (±0.01 g), and body weight gain was calculated accordingly. Feed intake was recorded on a replicate (cage) basis by offering feed ad libitum, replenished daily, and subtracting feed refusals collected at the same time each day. Results were expressed as g/bird/day dividing the total feed consumption per cage by the number of birds and the duration of the experimental period. Egg production (%) was calculated daily for each replicate as the ratio between the number of eggs collected and the number of birds per cage, multiplied by 100. Egg weight (g) was determined using an electronic scale, and egg mass (g/bird/day) was the egg mass was calculated by daily according to the previously measured egg production by the average egg weight. Feed conversion ratio was calculated as the ratio of feed intake to egg mass. The cage was considered the experimental unit for all performance variables.

### 2.5. Evaluation of Shell Integrity and Composition

To evaluate eggshell structural characteristics, a total of 150 eggs (10 eggs per replicate cage) were randomly selected from egg collections performed during the final 72 h of the experimental period. All measurements were conducted at the Egg Quality Laboratory of Selçuk University (Konya, Türkiye). The presence of external shell defects, including cracked or broken eggs, was recorded and expressed as a percentage of the total number of eggs evaluated. Eggshell breaking strength was measured at the blunt end of each egg using a calibrated force gauge (Egg Force Reader, Orka Food Tech, Herzliya, Israel), which applied increasing pressure until shell fracture occurred. Eggshell thickness was measured in micrometers at three locations (broad, equatorial and narrow ends) using a digital micrometer with a resolution of 0.01 mm (Mitutoyo, Kanagawa, Japan), and the mean value was calculated for each egg. To determine shell proportion, eggshells were manually cleaned, air-dried and weighed using a precision scale. Shell percentage was calculated as the ratio of shell weight to individual egg weight and expressed as a percentage.

### 2.6. Determination of Internal Quality of Eggs

Internal egg quality was evaluated using the same 150 eggs selected for eggshell quality analysis (10 eggs per replicate cage). Eggs were individually broken onto a clean, flat glass surface for measurements.

Albumen height and yolk height were measured using a digital height gauge, while albumen width, albumen length and yolk diameter were measured using a digital caliper with a precision of 0.01 mm (Mitutoyo, Japan). Based on these measurements, internal quality indices were calculated as follows: the albumen index was calculated as *[albumen height/((albumen width + albumen length)/2)] × 100*, and the yolk index was calculated as *[yolk height/yolk diameter] × 100*. Haugh units were calculated using the formula: *100 × log*_10_ *(albumen height + 7.57 − 1.7 × egg weight* ^0.37^*)*. Yolk color was assessed immediately after breaking the eggs. Color parameters L* (lightness), a* (redness) and b* (yellowness) were measured using a digital colorimeter (Minolta Chroma Meter CR-400, Minolta Co., Osaka, Japan). In addition, yolk color was evaluated visually using the Roche Color Fan. All measurements were performed carefully to preserve yolk integrity [1,2].

### 2.7. Quantification of Yolk Lipid Peroxidation and Antioxidant Capacity

Yolk antioxidant status was evaluated by determining malondialdehyde (MDA) concentration and antioxidant capacity using the DPPH radical-scavenging assay. A total of 24 freshly laid eggs (eight eggs per dietary treatment, collected from different replicate cages) were randomly selected on the 69th day of the experimental period. For antioxidant analyses, egg yolks were carefully separated from the albumen, rolled on filter paper to remove adhering albumen residues and homogenized thoroughly. Aliquots of the homogenized yolk (1.0 g) were then used for the determination of DPPH radical-scavenging activity and MDA concentration according to the procedures described below. All spectrophotometric measurements were performed in triplicate as technical replicates. Lipid peroxidation was assessed by measuring thiobarbituric acid-reactive substances (TBARS), following the method described by Kilic and Richards [30], with modifications reported by Sarmiento-García et al. [1]. Absorbance was recorded at 530 nm using a UV–visible spectrophotometer, and MDA concentration was calculated using a standard calibration curve. Results were expressed as µmol of MDA per kg of yolk. Yolk antioxidant capacity was determined using the DPPH (2,2-diphenyl-1-picrylhydrazyl) radical-scavenging assay, according to the procedure proposed by Sacchetti et al. [31] and adapted by Olgun et al. [32]. Absorbance was measured at 517 nm, and radical-scavenging activity was expressed as a percentage inhibition relative to the control sample.

### 2.8. Statistical Analysis

All statistical analyses were performed using SPSS software (version 31.0; IBM Corp., Armonk, NY, USA). Data were analyzed using a one-way analysis of variance (ANOVA), with dietary treatment as the fixed effect. For productive performance parameters, eggshell quality traits and internal egg quality variables, the cage was considered the experimental unit. For yolk antioxidant status variables (malondialdehyde concentration and DPPH radical-scavenging activity), individual eggs were considered the experimental unit. Percentage data (egg production, proportion of damaged eggs and DPPH inhibition values) were arcsine square-root transformed before analysis to meet the assumptions of normality. Normality of residuals was assessed using the Shapiro–Wilk test, and homogeneity of variances was evaluated using Levene’s test. When a significant treatment effect was detected, differences among means were compared using Tukey’s honestly significant difference (HSD) post hoc test. Results are presented as means with pooled standard error of the mean (SEM). Statistical significance was declared at *p* < 0.05.

## 3. Results

### 3.1. Performance

The effects of dietary supplementation with LRP on the productive performance of laying Japanese quail are presented in Table 3. Final body weight, body weight gain, egg production, egg weight, egg mass and feed conversion ratio were not affected by dietary treatment (*p* > 0.05).

In contrast, feed intake was significantly influenced by LRP (*p* < 0.001). Birds fed the diet supplemented with 1 g/kg lavender flower residue powder exhibited a significantly higher feed intake compared with the control group, whereas feed intake was significantly reduced in quail receiving 2 g/kg supplementation.

### 3.2. Eggshell Quality

The effects of dietary supplementation with LRP on eggshell quality parameters are presented in Table 4. The proportion of damaged eggs was significantly reduced in quail fed diets supplemented with LRP compared with the control group (*p* = 0.022), decreasing from 1.51% in the control group to 0.44% and 0.00% in birds receiving 1 and 2 g/kg of LRP, respectively. Eggshell breaking strength was markedly affected by dietary treatment (*p* < 0.001), increasing from 9.99 N in the control group to 11.48 N and 14.73 N in birds receiving 1 and 2 g/kg of LRP, respectively. In contrast, shell ratio and shell thickness were not influenced by dietary supplementation (*p* > 0.05).

### 3.3. Egg Internal Quality

The effects of dietary supplementation with LRP on internal egg quality parameters are presented in Table 5. Albumen index, yolk index, Haugh unit and yolk lightness (L*) and yellowness (b*) were not affected by dietary treatment (*p* > 0.05).

In contrast, yolk redness (a*) was significantly influenced by dietary supplementation (*p* = 0.003), with higher values observed in quail fed the diet supplemented with 1 g/kg LRP (6.03) compared with both the control group (5.19) and the 2 g/kg group (5.40). Similarly, yolk color evaluated using the Roche Color Fan was significantly affected by treatment (*p* = 0.001), reaching the highest score in the 1 g/kg group (7.88), followed by the 2 g/kg group (6.88), while the lowest value was observed in the control group (6.00).

### 3.4. Antioxidant Capacity of Yolk

The effects of dietary supplementation with LRP on yolk antioxidant status are presented in Table 6. Yolk antioxidant capacity, assessed by DPPH radical-scavenging activity, was significantly influenced by dietary treatment (*p* < 0.001). Birds fed the diet supplemented with 1 g/kg LRP showed higher DPPH-scavenging activity (14.64%) compared with both the control group (10.71%) and the 2 g/kg group (10.36%).

Yolk lipid peroxidation, evaluated by MDA concentration, was significantly reduced by lavender residue supplementation (*p* < 0.001). MDA levels decreased progressively with increasing supplementation level, from 5.285 µmol/kg in the control group to 4.112 µmol/kg and 3.513 µmol/kg in the 1 and 2 g/kg groups, respectively.

## 4. Discussion

Aromatic plants such as lavender are widely recognized as natural feed additives capable of modulating digestive processes and intestinal microbiota through their bioactive metabolites, potentially influencing animal performance [17]. However, in the present study, dietary supplementation with LRP did not affect egg production parameters or feed efficiency in laying quail. This absence of effect may be related to the nature of the tested material, as distillation residues contain lower concentrations of volatile compounds than essential oils, while retaining mainly phenolic constituents with antioxidant rather than growth-promoting activity [4]. Consistent with our findings, previous studies reported no significant effects on productive performance when lavender essential oil was supplemented to the diets of laying hens at 250 mg/kg [13] or broilers at inclusion levels ranging from 24 to 800 mg/kg [33,34]. In contrast, other studies have reported improvements in performance parameters following dietary lavender essential oil supplementation [11,12,16], while negative effects have also been observed at certain inclusion levels [35]. These contrasting results may reflect differences in the form of lavender used (essential oil vs. residue), inclusion level, animal species, production stage and experimental duration. Essential oils provide concentrated volatile compounds that may exert pronounced effects on feed palatability, digestive enzyme activity and microbial balance, whereas distillation residues are characterized by a milder phytochemical profile dominated by phenolic compounds. Consequently, lavender residue supplementation may exert a limited influence on productive performance while still modulating other physiological processes, such as oxidative status and egg quality, as observed in the present study.

In the present study, feed intake of laying quail increased at the supplementation level of 1 g/kg LRP, whereas a reduction was observed at the higher inclusion level of 2 g/kg. This non-linear response suggests a dose-dependent effect of lavender residue on feed palatability and voluntary intake. At low inclusion levels, phenolic compounds and other bioactive constituents may stimulate feed palatability and digestive activity, thereby increasing voluntary feed intake. However, at higher inclusion levels, the accumulation of aromatic and phenolic compounds with strong odor or bitter taste may reduce palatability, leading to a decrease in feed intake. Previous studies evaluating lavender essential oil supplementation have reported variable effects on feed intake, including increased consumption in broilers and laying quail [11,36], reduced intake in broilers [18], or inconsistent responses in laying quail [10]. These findings are partly consistent with the present results, indicating that lavender-derived products may exert both stimulatory and inhibitory effects on feed intake depending on dose and formulation. In contrast, other studies reported no significant effects of lavender essential oil supplementation on feed intake when administered through the diet or drinking water [12,13,35]. Differences across studies are likely related to differences in the form of lavender used, inclusion level and delivery matrix. Essential oils are highly concentrated sources of volatile compounds, whereas distillation residues contain lower levels of volatile and a higher proportion of phenolic compounds. At low inclusion levels, these compounds may enhance feed aroma and stimulate appetite, whereas higher inclusion levels may negatively affect palatability due to strong odor or bitter taste, ultimately reducing voluntary feed intake. This interpretation is supported by Windisch et al. [37], who reported that essential oils derived from aromatic plants can adversely affect feed intake when included at excessive levels due to their intense sensory properties.

Dietary supplementation with essential oils and other bioactive compounds derived from aromatic plants has been shown to positively modulate intestinal microflora, gastrointestinal pH and gut morphology in poultry [17,36,38,39]. These changes may enhance mineral utilization, particularly calcium, by improving its absorption and reducing fecal excretion [40,41,42]. Given that calcium is the primary structural component of the eggshell, such mechanisms are considered key drivers of improvements in eggshell quality following dietary supplementation with plant-derived bioactive compounds. In line with this concept, Taki et al. [43] reported that supplementation with 400 mg/kg lavender essential oil improved eggshell quality in laying hens. Similarly, we observed that dietary inclusion of LRP reduced the proportion of damaged eggs and significantly increased eggshell breaking strength in laying quail. Notably, these improvements occurred without changes in shell thickness or shell proportion, suggesting that LRP enhanced eggshell structural integrity rather than increasing calcium deposition. This response may reflect improved mineral organization or shell matrix integrity, resulting in greater mechanical strength at a similar thickness. Such dissociation between shell thickness and breaking strength has been previously reported and highlights that eggshell strength is influenced not only by shell quantity but also by shell microstructure and mineralization efficiency. However, the effects of lavender-derived products on eggshell quality reported in the literature are inconsistent. Torki et al. [13] observed no effects of lavender essential oil supplementation on shell thickness or shell strength in laying hens, while Özbilgin and Kara [11] reported similar findings in laying quail at inclusion levels ranging from 125 to 500 mg/kg. In contrast, Tatlı and Olgun [10] found that although lavender essential oil did not affect shell strength or the rate of damaged eggs, shell thickness increased at supplementation levels above 100 mg/kg. These discrepancies may be attributed to differences in the form of lavender used (essential oil versus distillation residue), inclusion level, species and physiological stage of the birds. Essential oils deliver concentrated volatile compounds, whereas distillation residues contain lower levels of volatiles and are richer in non-volatile phenolic compounds, which may exert subtler but more sustained effects on mineral metabolism and shell matrix formation. As suggested by Olgun and Yıldız [42], plant-derived bioactive compounds may stimulate enzymatic activity and amino acid availability involved in eggshell matrix formation, thereby improving shell strength without necessarily altering shell thickness. This mechanism may explain why LRP enhanced eggshell breaking strength and reduced egg damage while leaving shell thickness and shell proportion unchanged.

Yolk color is a key quality attribute influencing consumer preference, with increased pigmentation generally associated with higher product acceptance [2,3]. Here, we observed that, dietary supplementation with LRP resulted in an increase in yolk redness (a* value), which was statistically significant at the inclusion level of 1 g/kg. A similar pattern was observed for yolk color evaluated using the Roche Color Fan, with the highest score recorded in birds receiving 1 g/kg of lavender residue. Comparable effects on yolk color have been reported by Taki et al. [43], who observed improved yolk pigmentation following supplementation with 400 mg/kg lavender essential oil. However, other studies found no significant effects of lavender essential oil supplementation on yolk color in laying hens or quail at inclusion levels ranging from 125 to 500 mg/kg [11,13]. These inconsistencies suggest that the impact of lavender-derived products on yolk pigmentation is highly dependent on the form of lavender used and its phytochemical composition. Carotenoids are the primary pigments responsible for yolk color, and green plant materials and their by-products are recognized as important dietary sources of these compounds [44]. Unlike essential oils, which mainly contain volatile compounds and are largely devoid of carotenoids, lavender flower distillation residues retain non-volatile plant constituents, including pigments and phenolic compounds. Therefore, the observed increase in yolk a* value and Roche color score in the present study may be attributed to the carotenoid content of the lavender flower residue powder. This interpretation is supported by the present data, which showed a significant increase in these color parameters at the 1 g/kg inclusion level. The lack of a further increase in yolk pigmentation at the higher inclusion level (2 g/kg) may reflect a saturation effect in pigment deposition or differences in pigment bioavailability at higher dietary concentrations. Overall, these findings suggest that LRP may represent a valuable plant-derived by-product for enhancing yolk color when included at moderate dietary levels. Moreover, the effects observed in the present study are consistent with those reported for other plant-derived agricultural by-products used in laying poultry nutrition. Studies evaluating purple carrot [1], lemon peel [2] and red cabbage [3] processing residues have similarly reported improvements in yolk pigmentation, largely attributed to their content of phenolic compounds and natural pigments. In this context, the responses observed with LRP fit within a broader pattern reported for functional agro-industrial by-products, supporting the relevance of this approach for enhancing a key quality attribute influencing consumer preference.

Lavender is widely recognized for its antioxidant properties, which are attributed to the presence of bioactive compounds such as phenolics and terpenoids [15,17,18,36]. In particular, linalool, one of the major bioactive constituents of lavender, exhibits antioxidant activity comparable to that of ascorbic acid by donating hydrogen atoms and scavenging free radicals, including DPPH radicals [45]. Our results indicate that yolk antioxidant capacity, assessed by DPPH radical-scavenging activity, was significantly increased by dietary supplementation with LRP at the inclusion level of 1 g/kg. The absence of further improvement in yolk antioxidant indices at the higher inclusion level (2 g/kg) compared with 1 g/kg may reflect a saturation of antioxidant deposition or utilization within the yolk. At moderate inclusion levels, bioactive compounds from LRP may be efficiently absorbed and incorporated, whereas higher dietary concentrations may exceed physiological requirements, resulting in limited additional benefits. Moreover, reduced bioavailability or interactions among phenolic compounds at higher doses cannot be excluded and may contribute to the non-linear antioxidant response observed. Evidence regarding the effects of lavender-derived products on egg yolk antioxidant status in laying birds is limited. Özbilgin and Kara [11] reported no changes in yolk MDA levels following dietary supplementation with lavender essential oil. In contrast, the current study demonstrated a significant, dose-dependent reduction in yolk MDA concentration with increasing dietary levels of LRP, indicating a protective effect against lipid peroxidation in egg yolk. The discrepancy between both studies may be related to differences in the form of lavender used and the target tissue analyzed. While essential oils primarily contain volatile compounds, LRP retain non-volatile phenolic constituents that may exert sustained antioxidant effects within lipid-rich matrices such as egg yolk. Although most available mechanistic evidence derives from studies conducted in meat-type poultry, these studies consistently show that lavender-derived compounds enhance antioxidant defense systems and reduce lipid peroxidation in biological tissues. In this context, increased superoxide dismutase (SOD) activity in broiler meat [18,33,36], as well as reduced MDA value and elevated total antioxidant capacity, SOD and catalase (CAT) activities in serum following dietary supplementation [15] or administration through drinking water [14], have been reported. Furthermore, Barbarestani et al. [17] observed improvements in SOD and glutathione peroxidase (GSH-Px) activities in serum and liver of broilers fed diets supplemented with lavender essential oil. These findings are consistent with the enhanced antioxidant capacity observed in egg yolk in the present study. MDA is a well-established secondary product of lipid peroxidation and is commonly used as an indicator of oxidative damage to cell membranes. In the current research, increasing levels of LRP in the diet resulted in a significant, dose-dependent reduction in yolk MDA concentration, indicating a protective effect against lipid peroxidation. Collectively, the present findings suggest that the bioactive compounds present in LRP are effective in enhancing yolk antioxidant capacity and reducing lipid peroxidation, thereby contributing to improved oxidative stability of egg.

The findings of the current research directly address the research questions outlined in the Introduction by demonstrating that LRP can be used as a functional feed additive in laying quails, with measurable effects on egg quality traits and yolk oxidative stability.

## 5. Conclusions

This study suggests that LRP can be used as a functional feed ingredient in laying quail diets, with potential benefits for eggshell integrity, yolk color and yolk antioxidant stability, without adversely affecting productive performance. These findings indicate that lavender processing by-products may represent sustainable alternatives to conventional feed additives.

From a societal and environmental perspective, the valorization of lavender distillation residues aligns with circular economy principles by reducing agro-industrial waste and promoting the reuse of plant by-products with added functional value. From a practical standpoint, moderate dietary inclusion levels (around 1 g/kg) appear to be sufficient to obtain beneficial effects on egg quality traits. In contrast, higher levels may not provide additional advantages.

Nevertheless, the present study has certain limitations. In particular, direct measurements of intestinal mineral absorption, detailed carotenoid and phenolic profiling of the lavender residue, and antioxidant enzyme activities in birds were not assessed, which would allow a more precise elucidation of the underlying mechanisms. Despite these limitations, the results provide novel evidence that lavender flower distillation residue may influence eggshell quality, yolk pigmentation and yolk oxidative stability in laying quails. Future studies should therefore characterize the phytochemical composition of lavender distillation residues in greater detail, evaluate their physiological effects across different poultry species and production stages, and investigate their interactions with mineral metabolism and antioxidant defense systems. Long-term trials under commercial production conditions would also help confirm the robustness and practical applicability of these findings.

## Figures and Tables

**Table 1:** DPPH and total phenolic content of LRP.

Parameters	Level
DPPH, % reducing	75.42
Total phenolic content, mg GAE/g	2069.96

DPPH: 2,2-diphenyl-1-picrylhydrazyl, GAE: Gallic acid equivalent.

**Table 2:** Basal diet and its nutrient contents.

Ingredients	g/kg	Nutrients	g/kg
Corn	537.6	Metabolic energy, kcal/kg	2899
Soybean meal	350.0	Moisture ^2^	128.3
Soybean oil	37.3	Crude protein ^2^	199.7
Limestone	56.1	Crude fat ^2^	58.9
Dicalcium phosphate	11.4	Crude fiber ^2^	28.3
Salt	3.5	Calcium ^2^	25.0
Premix ^1^	1.0	Total phosphorus ^2^	6.4
DL-methionine	2.1	Available phosphorus	3.5
Toxin binder	1.0	Lysine	11.0
		Methionine	4.5
		Methionine + cystine	8.6

^1^ The premix provides 13,000 IU vitamin A (trans-retinol); 5000 IU vitamin D_3_ (cholecalciferol); 80 mg vitamin E (α-tocopherol acetate); 4 mg vitamin K_3_ (menadione); 5 mg thiamine; 9 mg riboflavin; 0.35 mg biotin; 300 mg choline chloride; 70 mg niacin; 25 mg pantothenic acid; 5 mg pyridoxin; 2.5 mg folic acid; 0.02 mg cyanocobalamin mg; 16 mg copper (CuSO_4_∙5H_2_O); 1.25 mg; 20 mg iron (FeSO_4_∙7H_2_O); 120 mg manganese (MnSO_4_∙H_2_O); 0.30 mg selenium (NaSeO_3_); 120 mg zinc (ZnO) in 1 kg of diet. ^2^ Analyzed values.

**Table 3:** The effect of the addition of lavender flower residue powder to the diet on the performance in laying quails *(n* = 25).

Parameters	Lavender Flower Distillation Residue Powder, g/kg			SEM	*p*-Values
	0	1	2		
Initial body weight, g	271.9	272.2	268.5	1.37	0.503
Final body weight, g	280.7	289.2	285.7	1.95	0.213
Body weight gain, g	8.88	17.00	17.25	2.035	0.163
Egg production, %	91.68	93.57	92.61	0.851	0.369
Egg weight, g	13.11	13.35	12.59	0.200	0.303
Egg mass, g/bird/d	12.06	12.51	11.65	0.263	0.439
Feed intake, g/bird/d	34.54 ^b^	36.49 ^a^	33.24 ^c^	0.405	<0.001
Feed conversion ratio	2.902	2.929	2.854	0.0504	0.849

SEM: standard error of the mean. ^a,b,c^: Means with different superscripts in the same row were significantly different at *p* < 0.05.

**Table 4:** The effect of the addition of lavender flower distillation residue powder to the diet on the eggshell quality (*n* = 50) in laying quails.

Parameters	Lavender Flower Distillation Residue Powder, g/kg			SEM	*p*-Values
	0	1	2		
Damaged eggs rate, %	1.51 ^a^	0.44 ^b^	0.00 ^b^	0.247	0.022
Shell strength, N	9.99 ^c^	11.48 ^b^	14.73 ^a^	0.582	<0.001
Shell ratio, %	8.09	8.15	8.03	0.098	0.906
Shell thickness, µm	216.3	234.8	232.9	4.30	0.155

SEM: standard error of the mean. ^a,b,c^: Means with different superscripts in the same row were significantly different at *p* < 0.05.

**Table 5:** The effect of the addition of lavender flower distillation residue powder to the diet on the internal quality of egg (*n* = 50) in laying quails.

Parameters	Lavender Flower Distillation Residue Powder, g/kg			SEM	*p*-Values
	0	1	2		
Albumen index	2.33	2.60	2.21	0.117	0.429
Yolk index	50.10	48.70	47.16	0.861	0.410
Haugh Unit	83.59	87.28	84.00	1.063	0.323
L*	59.28	58.30	58.95	0.480	0.729
a*	5.19 ^b^	6.03 ^a^	5.40 ^b^	0.121	0.003
b*	36.08	35.82	37.27	0.468	0.436
Roche Color Fan	6.00 ^c^	7.88 ^a^	6.88 ^b^	0.251	0.001

SEM: standard error of the mean; L*: Lightness, a*: Redness, b*: Yellowness. ^a,b,c^: Means with different superscripts in the same row were significantly different at *p* < 0.05.

**Table 6:** The effect of the addition of lavender flower distillation residue powder to the diet on the antioxidant capacity of yolk (*n* = 8) in laying quails.

Parameters	Lavender Flower Distillation Residue Powder, g/kg			SEM	*p*-Values
	0	1	2		
DPPH, % reducing	10.71 ^b^	14.64 ^a^	10.36 ^b^	0.454	<0.001
MDA, µmol/kg	5.285 ^a^	4.112 ^b^	3.513 ^c^	0.1904	<0.001

DPPH: 2,2-diphenyl-1-picrylhydrazyl, MDA: Malondialdehyde; SEM: Standard error of the mean. ^a,b,c^: Means with different superscripts in the same row were significantly different at *p* < 0.05.

## Data Availability

The data analyzed for the study are available from the corresponding author upon reasonable request.

## Abbreviations

DPPH	2,2-diphenyl-1-picrylhydrazyl
MDA	Malondialdehyde
TBARS	Thiobarbituric acid-reactive substances
SOD	Superoxide dismutase
CAT	Catalase
GSH-Px	Glutathione peroxidase

## References

[1] Sarmiento-Garcia A., Olgun O., Kılınç G., Sevim B., Gökmen S.A. (2023). The use of purple carrot powder in the diet of laying quails improved some egg quality characteristics, including antioxidant capacity. Trop. Anim. Health Prod..

[2] Sarmiento-Garcia A., Olgun O., Gül E.T., Kılınç G., Yıldız A. (2024). The potential of lemon peel powder as an additive in layer quails (*Coturnix coturnix Japonica*): An experimental study. An. Acad. Bras. Ciênc..

[3] Gümüş E., Sevim B., Sırakaya S., İriş C., Kahraman O., Sarmiento-Garcia A. (2025). Evaluation of the effect of red cabbage waste on performance, egg quality, and yolk oxidative stability of laying Japanese quails (*Coturnix coturnix Japonica*). Trop. Anim. Health Prod..

[4] Başer E., Sevim B., Akbulut N.K., Cufadar Y., Halıcı İ., Kal Y., Tekik H., Gültekin M., Sarmiento-García A. (2025). Thyme (*Thymus vulgaris L*.) essential oil supplementation: Impact on performance, egg quality, serum, and faecal mineral content in laying partridges. Span. J. Agric. Res..

[5] Adaszyńska-Skwirzyńska M., Szczerbińska D. (2019). The effect of lavender (*Lavandula angustifolia*) essential oil as a drinking water supplement on the production performance, blood biochemical parameters, and ileal microflora in broiler chickens. Poult. Sci..

[6] Radi F.Z., Zekri N., Drioiche A., Zerkani H., Boutakiout A., Bouzoubaa A., Zair T. (2022). Volatile and non-volatile chemical compounds and biological power of the genus Lavandula: Case of two Moroccan Lavenders *Lavandula angustifolia* Mill. (cultivated lavender) and *Lavandula pedunculata* (Mill.) Cav. (*Spontaneous Lavender*). Egypt. J. Chem..

[7] Betlej I., Andres B., Cebulak T., Kapusta I., Balawejder M., Jaworski S., Lange A., Kutwin M., Pisulewska E., Kidacka A. (2023). Antimicrobial properties and assessment of the content of bioactive compounds *Lavandula angustifolia* Mill. cultivated in Southern Poland. Molecules.

[8] Gallego M.G., Gordon M.H., Segovia F.J., Skowyra M., Almajano M.P. (2013). Antioxidant properties of three aromatic herbs (rosemary, thyme and lavender) in oil-in-water emulsions. J. Am. Oil Chem. Soc..

[9] Andrys D., Kulpa D., Grzeszczuk M., Bihun M., Dobrowolska A. (2017). Antioxidant and antimicrobial activities of Lavandula angustifolia Mill. field-grown and propagated in vitro. Folia Hortic..

[10] Tatlı Y., Olgun O. (2021). Effect of supplementation of lavender essential oil to the diet on performance, egg quality and serum parameters in laying quails. J. Bahri Dagdas Anim. Res..

[11] Özbilgin A., Kara K. (2023). Effect of adding lavender oil to laying quail diets on performance, egg quality, oxidative status, and fatty acid profile. Trop. Anim. Health Prod..

[12] Alaşahan S., Önel S.E., Eraslan–Şakar A., Köse S.İ., Kutlu T., Özcan C. (2025). Effect of *Lavandula stoechas* and hydrogen peroxide on quails in terms of egg production, liver function, blood parameters, and gene expression. Rev. Cient.-Fac. Cien. Vet..

[13] Torki M., Mohebbifar A., Mohammadi H. (2021). Effects of supplementing hen diet with *Lavandula angustifolia* and/or *Mentha spicata* essential oils on production performance, egg quality and blood variables of laying hens. Vet. Med. Sci..

[14] Adaszyńska-Skwirzyńska M., Szczerbińska D., Zych S. (2021). The use of lavender (*Lavandula angustifolia*) essential oil as an additive to drinking water for broiler chickens and its in vitro reaction with enrofloxacin. Animals.

[15] Amer S.A., Abdel-Wareth A.A.A., Gouda A., Saleh G.K., Nassar A.H., Sherief W.R.I.A., Albogami S., Shalaby S.I., Abdelazim A.M., Abomughaid M.M. (2022). Impact of dietary lavender essential oil on the growth and fatty acid profile of breast muscles, antioxidant activity, and inflammatory responses in broiler chickens. Antioxidants.

[16] Çolak A., Yıldız A. (2024). The effect of lavender essential oil on performance, carcass characteristics and serum parameters of broilers. J. Hell. Vet. Med. Soc..

[17] Barbarestani S.Y., Jazi V., Mohebodini H., Ashayerizadeh A., Shabani A., Toghyani M. (2020). Effects of dietary lavender essential oil on growth performance, intestinal function, and antioxidant status of broiler chickens. Livest. Sci..

[18] Adil S., Banday M.T., Hussain S.A., Wani M.A., Khan A.A., Sheikh I.U., Afzal Y., Kashoo Z., Shah S., Shilbayeh S.A.R. (2025). Modulation of performance, meat quality and gut health of broiler chicken in response to dietary nanoencapsulated lavender essential oil. Sci. Rep..

[19] Hedayati S., Tarahi M., Madani A., Mazloomi S.M., Hashempur M.H. (2025). Towards a greener future: Sustainable innovations in the extraction of lavender (*Lavandula* spp.) essential oil. Foods.

[20] Zheljazkov V.D., Cantrell C.L., Astatkie T., Jeliazkova E. (2013). Distillation time effect on lavender essential oil yield and composition. J. Oleo Sci..

[21] Lesage-Meessen L., Bou M., Sigoillot J.C., Faulds C.B., Lomascolo A. (2015). Essential oils and distilled straws of lavender and lavandin: A review of current use and potential application in white biotechnology. Appl. Microbiol. Biotechnol..

[22] Aprotosoaie A.C., Gille E., Trifan A., Luca V.S., Miron A. (2017). Essential oils of Lavandula genus: A systematic review of their chemistry. Phytochem. Rev..

[23] Lesage-Meessen L., Bou M., Ginies C., Chevret D., Navarro D., Drula E., Bonnin E., del Río R.C., Odinot E., Bisotto A. (2018). Lavender-and lavandin-distilled straws: An untapped feedstock with great potential for the production of high-added value compounds and fungal enzymes. Biotechnol. Biofuels.

[24] Slavov A.M., Karneva K.B., Vasileva I.N., Denev P.N., Denkova R.S., Shikov V.T., Manolova M.N., Lazarova Y.L., Ivanova V.N. (2018). Valorization of lavender waste—Obtaining and characteristics of polyphenol rich extracts. Food Sci. Appl. Biotechnol..

[25] Marovska G., Vasileva I., Petkova N., Ognyanov M., Gandova V., Stoyanova A., Merdzhanov P., Simitchev A., Slavov A. (2022). Lavender (*Lavandula angustifolia* Mill.) industrial by-products as a source of polysaccharides. Ind. Crops Prod..

[26] Kara K., Yilmaz Öztaş S., Baytok E. (2025). In vitro ruminal metagenomic profiles and ruminal fermentation variables of aromatic plant pulps. Vet. Med. Sci..

[27] National Research Council (NRC) (1994). Nutrient Requirements of Poultry.

[28] Singleton V.L., Rossi J.A. (1965). Colorimetry of total phenolics with phosphomolybdic-phosphotungstic acid reagents. Am. J. Enol. Vitic..

[29] AOAC (2006). Official Methods of Analysis Association.

[30] Kilic B., Richards M.P. (2003). Lipid oxidation in poultry döner kebab: Pro-oxidative and anti-oxidative factors. J. Food Sci..

[31] Sacchetti G., Maietti S., Muzzoli M., Scaglianti M., Manfredini S., Radice M., Bruni R. (2005). Comparative evaluation of 11 essential oils of different origin as functional antioxidants, antiradicals and antimicrobials in foods. Food Chem..

[32] Olgun O., Gül E.T., Kılınç G., Yıldız A., Çolak A., Sarmiento-García A. (2022). Performance, egg quality, and yolk antioxidant capacity of the laying quail in response to dietary choline levels. Animals.

[33] Küçükyılmaz K., Kıyma Z., Akdağ A., Çetinkaya M., Atalay H., Ateş A., Gürsel F.E., Bozkurt M. (2017). Effect of lavender (*Lavandula stoechas*) essential oil on growth performance, carcass characteristics, meat quality and antioxidant status of broilers. S. Afr. J. Anim. Sci..

[34] Mokhtari S., Rahati M., Seidavi A., Haq Q.M.I., Kadim I., Laudadio V., Tufarelli V. (2018). Effects of feed supplementation with lavender (*Lavandula angustifolia*) essence on growth performance, carcass traits, blood constituents and caecal microbiota of broiler chickens. Eur. Poult. Sci..

[35] Gümüş İ.H., Şenses E., Sözcü A. (2024). Improving egg production, quality and eggshell hygiene by supplementation of sage (*Salvia officinalis*) and lavender (*Lavandula angustifolia*) essential oils. J. Anim. Prod..

[36] Salarmoini M., Salajegheh A., Salajegheh M.H., Afsharmanesh M. (2019). The effect of lavender (*Lavandula angustifolia*) extract in comparison to antibiotic on growth performance, intestinal morphology, ileal microflora, antioxidant status and meat quality of broilers. Iran. J. Appl. Anim. Sci..

[37] Windisch W., Schedle K., Plitzner C., Kroismayr A. (2008). Use of phytogenic products as feed additives for swine and poultry. J. Anim. Sci..

[38] Hassan M.A., Yusuf M.S., Kilany O.E., Khalil H.A., Hanafy A.M., Hassan A.M. (2015). Evaluation of essential oil mixture overuse on gut health and some immune parameters in laying Japanese quail (*Coturnix Coturnix japonica*). Int. Proc. Chem. Biol. Environ. Eng..

[39] Laghouati O., Arbouche F., Arbouche Y. (2020). Effects of using essential oil of *Lavandula stoechas* in quail feed on growth performance, carcass characteristics, meat quality, and health status. Vet. World.

[40] Amad A.A., Manner K., Wendler K.R., Neumann K., Zentek J. (2011). Effects of a phytogenic feed additive on growth performance and ileal nutrient digestibility in broiler chickens. Poult. Sci..

[41] Mountzouris K.C., Paraskevas V., Tsirtsikos P., Palamidi I., Steiner T., Schatzmayr G., Fegeros K. (2011). Assessment of a phytogenic feed additive effect on broiler growth performance, nutrient digestibility and caecal microflora composition. Anim. Feed. Sci. Technol..

[42] Olgun O., Yıldız A.Ö. (2014). Effect of dietary supplementation of essential oils mixture on performance, eggshell quality, hatchability, and mineral excretion in quail breeders. Environ. Sci. Pollut. Res..

[43] Taki A., Salari S., Boujarpour M., Sari M., Taghizadeh M. (2015). Effects of feeding various levels of *Lavandula stoechas* essence on quantitative and qualitative characteristics of egg, some blood parameters, and morphological changes of ovary in laying hens. Iran. Vet. J..

[44] Zurak D., Slovenec P., Janječić Z., Bedeković X.D., Pintar J., Kljak K. (2022). Overview on recent findings of nutritional and non-nutritional factors affecting egg yolk pigmentation. World’s Poult. Sci. J..

[45] Jabir M.S., Taha A.A., Sahib U.I. (2018). Antioxidant activity of linalool. Eng. Technol. J..

